# Arthroscopic Superior Capsule Reconstruction Using Hamstring Allograft in V‐Shaped Configuration

**DOI:** 10.1002/atn2.70212

**Published:** 2026-08-14

**Authors:** Hernando Canal Torres, Valeria Latorre Jaramillo, Luis Alfredo Moreno, Rodrigo Lopez, Gustavo Chavarro, Ricardo González, Fabio Restrepo

**Affiliations:** ^1^ Clínica Reina Sofía Bogotá Colombia

## Abstract

Massive irreparable rotator cuff tears remain a challenge for the orthopaedic surgeon a great spectrum from conservative to surgical procedures have been described. Superior capsular reconstruction is an option in active young patients with massive irreparable rotator cuff tears without arthritis, which reduce the humeral head superior migration, restoring the glenohumeral fulcrum while maintaining stable force couples. However, it is a demanding high‐cost procedure with substantial retear rate. A recent report has been published, showing an open procedure using semitendinosus autograft. In this technique, we describe an all‐arthroscopic superior capsular reconstruction using hamstring allograft in V‐shaped configuration. We believe our technique can be technically reproducible at a lower cost with excellent clinical outcomes.

## Graphical abstract

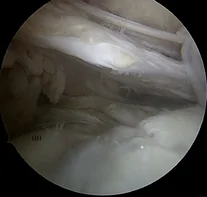

**VIDEO 1:** This video shows the surgical steps to superior capsule reconstruction using hamstring allograft in a right shoulder in a beach chair position. After visualizing the remaining rotator cuff from a posterolateral viewing portal, debridement of the superior glenoid rim is done. The first pilot hole in the center of the superior rim is drilled to place the first knotless anchor. The allograft if prepared on a separate auxiliary table with a Krackow stitch on each end of the allograft and a high‐strength polyethylene suture (FiberWire, Arthrex, Naples, FL) in the middle of the graft creating a vertex. The allograft is introduced through the lateral portal and is fixed to the superior glenoid rim. From a posterior viewing portal the fixation to the greater tuberosity is done anterolateral and posterolateral with adequate tension with the patient in beach‐chair position, with the operative arm maintained in longitudinal traction at 40° of forward elevation and 40° of abduction. Finally, the superior capsule reconstruction is visualized in V configuration. Video content can be viewed at https://doi.org/10.1002/atn2.70212.

Massive irreparable rotator cuff tears (MIRCTs) represent a complex and challenging pathology for shoulder surgeons. The definition of MIRCT is variable; 5 or more cm tears in the coronal plane described by Cofield,^1^ a tear involving 2 or more rotator cuff tendons^2^ tear patterns and their clinical significance described by Collin et al.^3^ Irreparability takes other factors in consideration such as: fatty infiltration greater than grade 3 or 4 according to Goutallier, magnitude of retraction, and the presence of chronic pseudo paralysis.^4^ Many approaches have been described to treat this pathology, both surgical and nonsurgical. The indication on surgical management depends on the functionality of the shoulder and grade of rotator cuff arthropathy. The wide variety of techniques include partial repair, superior capsular reconstruction, and tendon transfers. We describe a superior capsular reconstruction variation using a hamstring allograft with a V‐shaped configuration.

## SURGICAL TECHNIQUE

**FIGURE 1:**
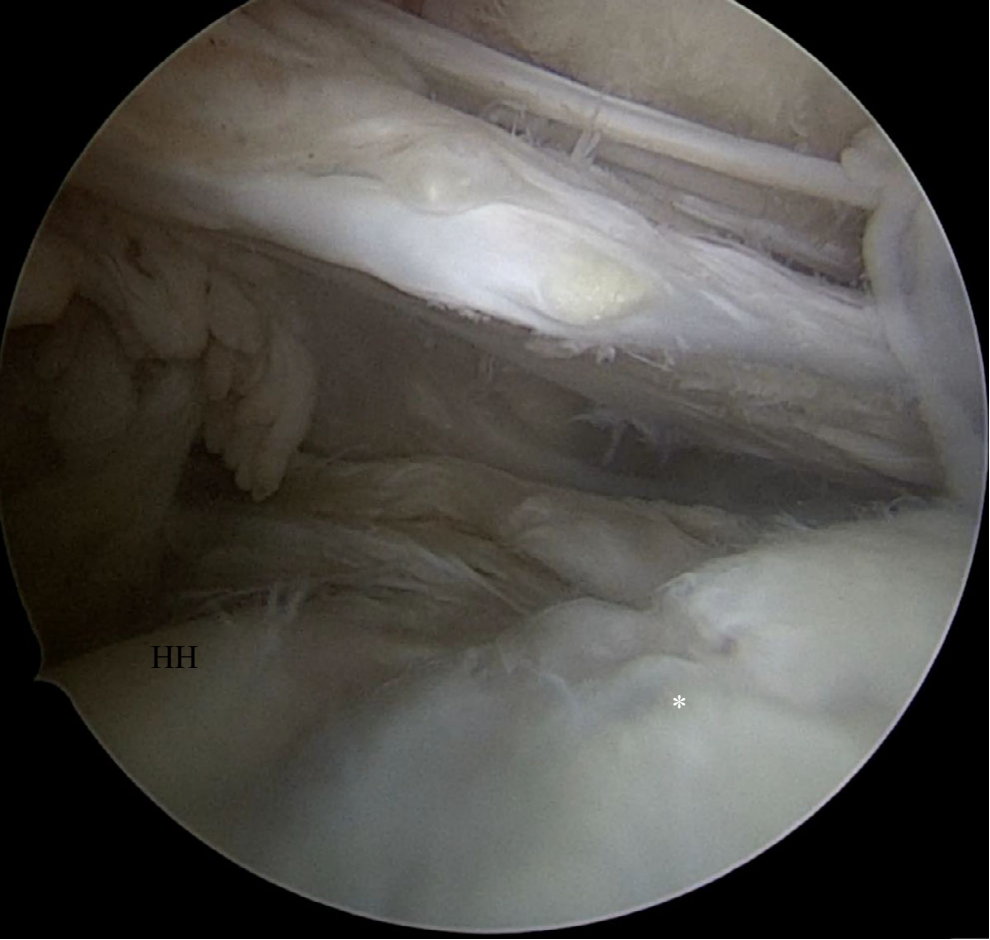
Intraoperative view of right shoulder in beach chair position using a 30° lens from a posterolateral viewing portal to visualize the massive rotator cuff tear. *Rotator cuff footprint. (HH, humeral head.)

Our indication for superior capsular reconstruction using hamstring allograft include patients with a MIRCT, Patte grade 3 retraction in coronal plane, Goutallier grade 3 or 4 fatty infiltration, Hamada 2 rotator cuff arthropathy, full passive and active range of motion, competent external rotators, and absence or inability to repair with the long head of the biceps. These criteria must be documented by magnetic resonance imaging. The surgery is performed under general anesthesia and inter‐scalene block with the patient placed in beach chair position. The initial arthroscopic exploration is executed using posterior, posterolateral, Neviaser, and anterolateral portals, with a 30° lens in order to better characterize the massive tear and evaluate the integrity of the subscapularis tendon (Figure 1, Video 1). In the presence of a partial reparable subscapularis tear, it must be repaired beforehand.

### Graft Preparation

**FIGURE 2:**
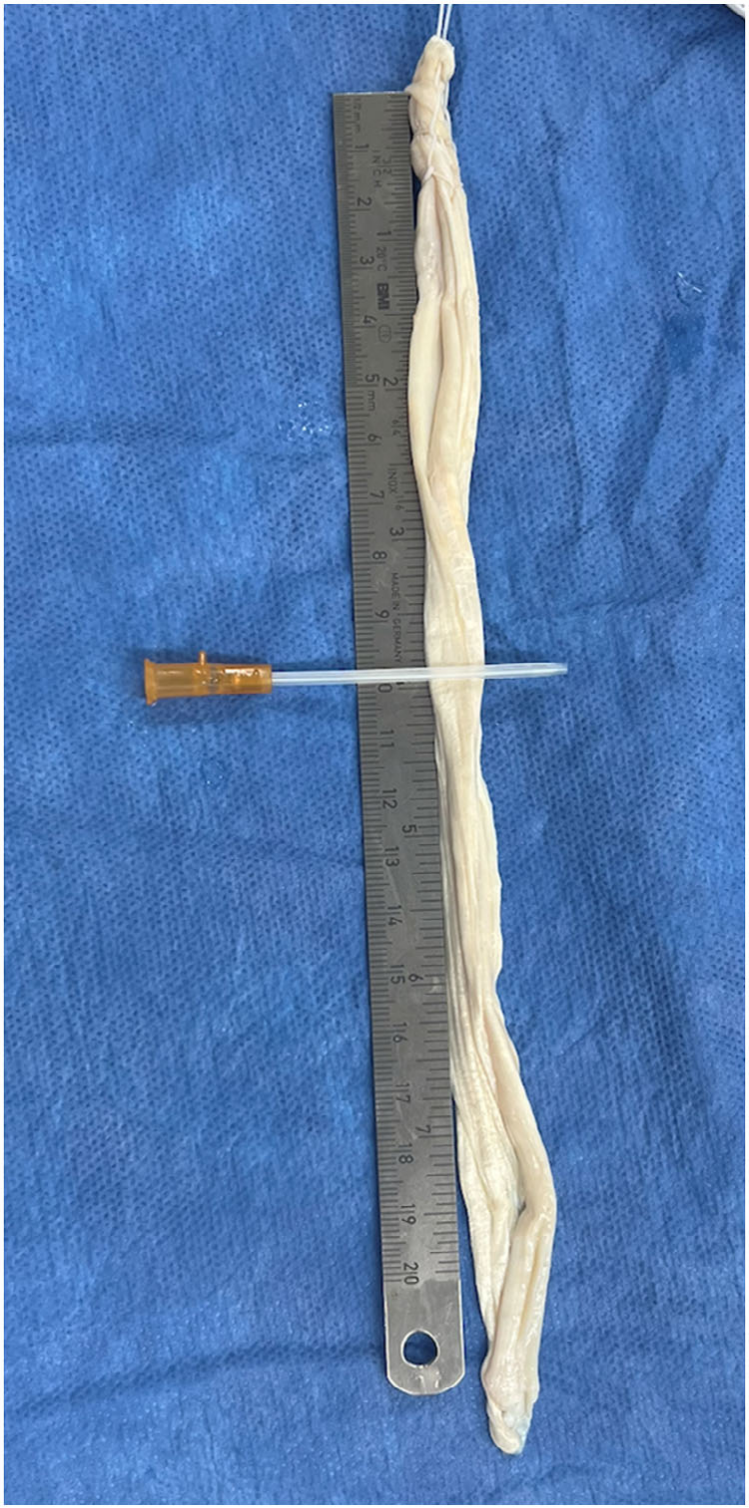
Intraoperative view of preparation of hamstring allograft in a separate auxiliary surgical table with optimal 10 to 12 cm long and 8 mm width.

**FIGURE 3:**
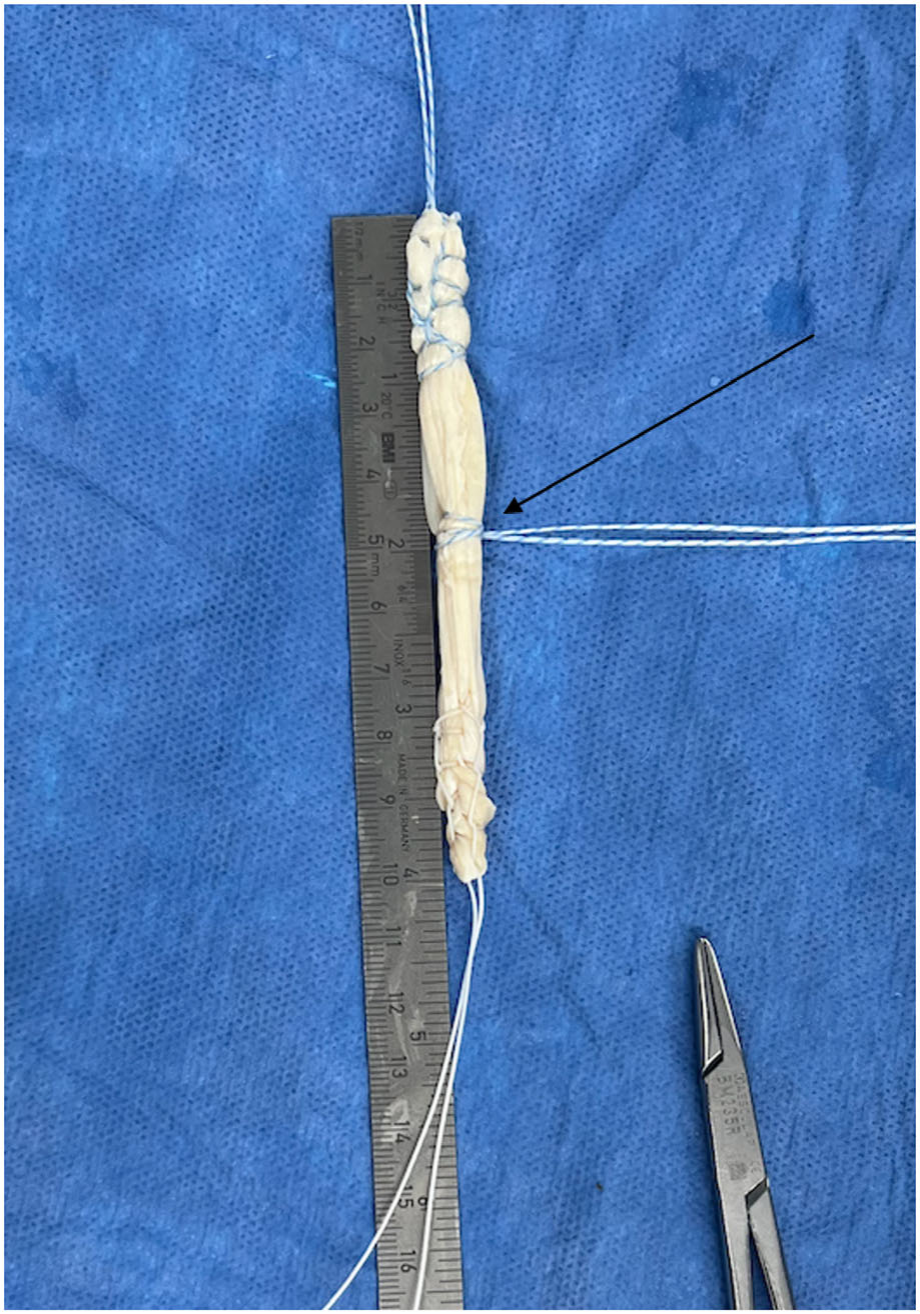
Intraoperative view of preparation of hamstring allograft in a separate auxiliary surgical table folded into two 5 to 6 cm segments by passing a high‐strength polyethylene suture (FiberWire, Arthrex, Naples, FL) in the middle of the graft creating a vertex. Arrow: midpoint of the allograft.

**FIGURE 4:**
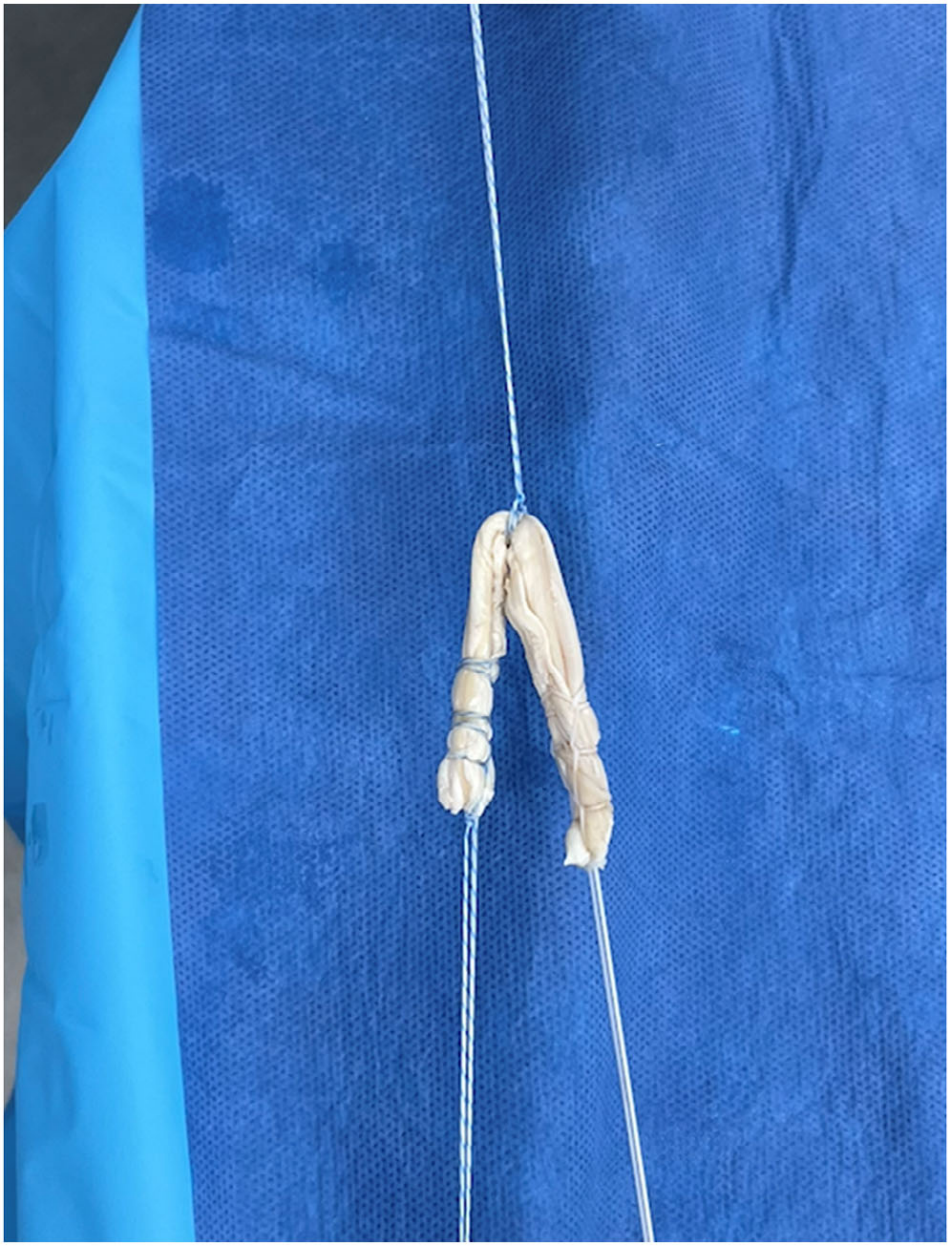
Intraoperative view of preparation of hamstring allograft in a separate auxiliary surgical table, the ends of each strand of the allograft are repaired with a polyethylene suture using a Krackow stitch to facilitate its manipulation.

The hamstring allograft must have a dimension of 8 mm in thickness and 10 to 12 cm in length after preparation (Figure 2). The graft must be folded into two 5 to 6 cm segments by passing a high‐strength polyethylene suture (FiberWire, Arthrex, Naples, FL) in the middle of the graft creating a vertex. (Figure 3). The ends of each strand are repaired with a polyethylene suture using a Krackow stitch to facilitate its manipulation (Figure 4). Use different color sutures preferably.

### Glenoid and Tuberosity Preparation

The rest of the rotator cuff is evaluated on the subacromial space through the posterolateral portal. We debride the soft tissue on the superior glenoid rim using radiofrequency and shaver. Then, the bone on the greater tuberosity is fully exposed using shaver and radiofrequency.

### Graft Implantation

**FIGURE 5:**
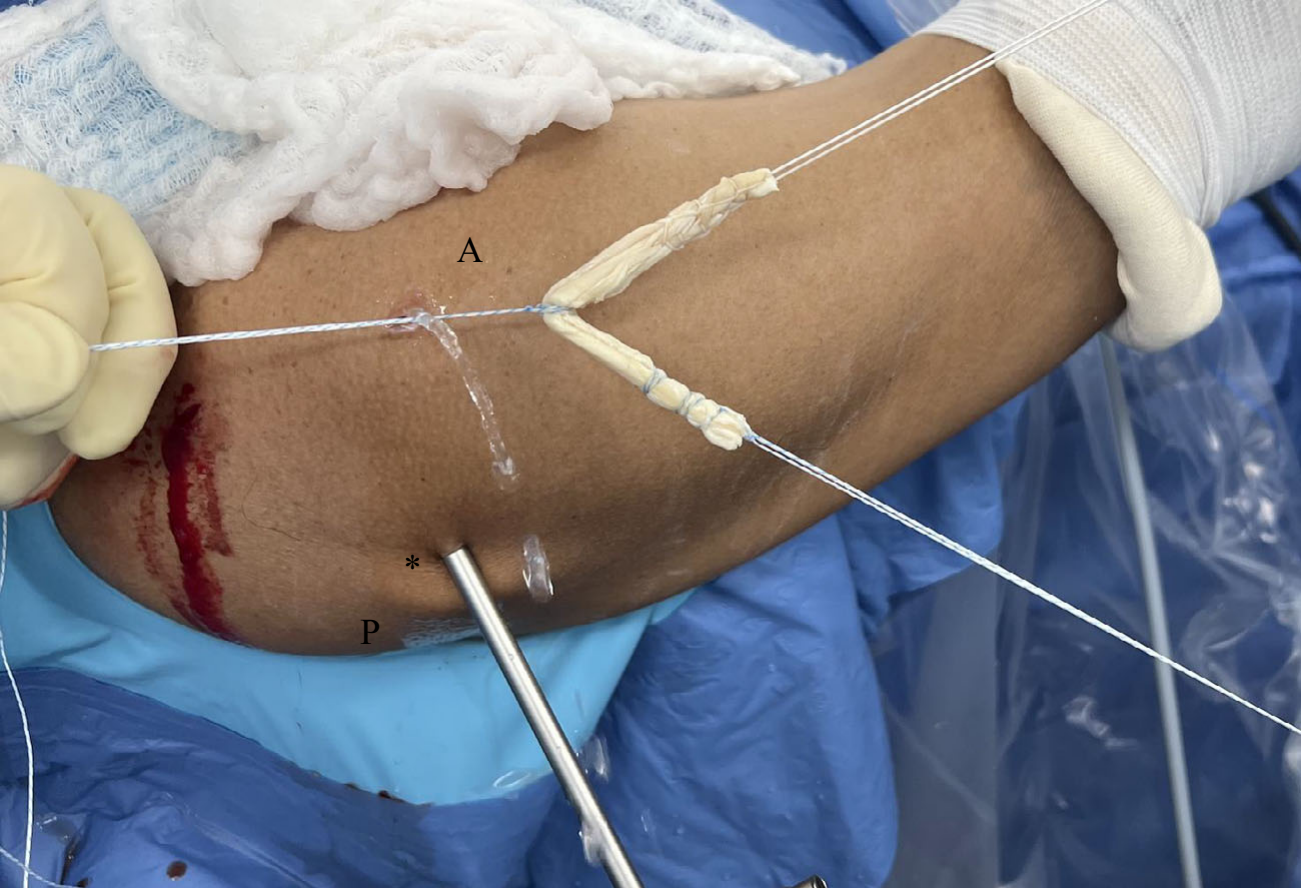
Intraoperative view of right shoulder in beach chair position, viewing from the posterolateral portal, insertion of the allograft through the lateral portal. *Posterolateral viewing portal. (A: anterior; P: posterior).

**FIGURE 6:**
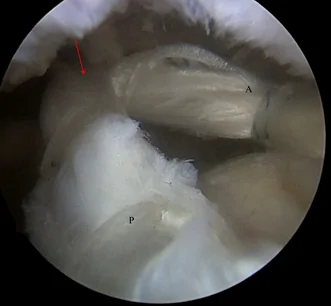
Intraoperative view of right shoulder in beach chair position from posterolateral portal with final construct in a V‐shaped configuration acting as a mechanical barrier to prevent superior humeral head migration. (A: anterior; P: posterior; arrow: glenoid border).

**FIGURE 7:**
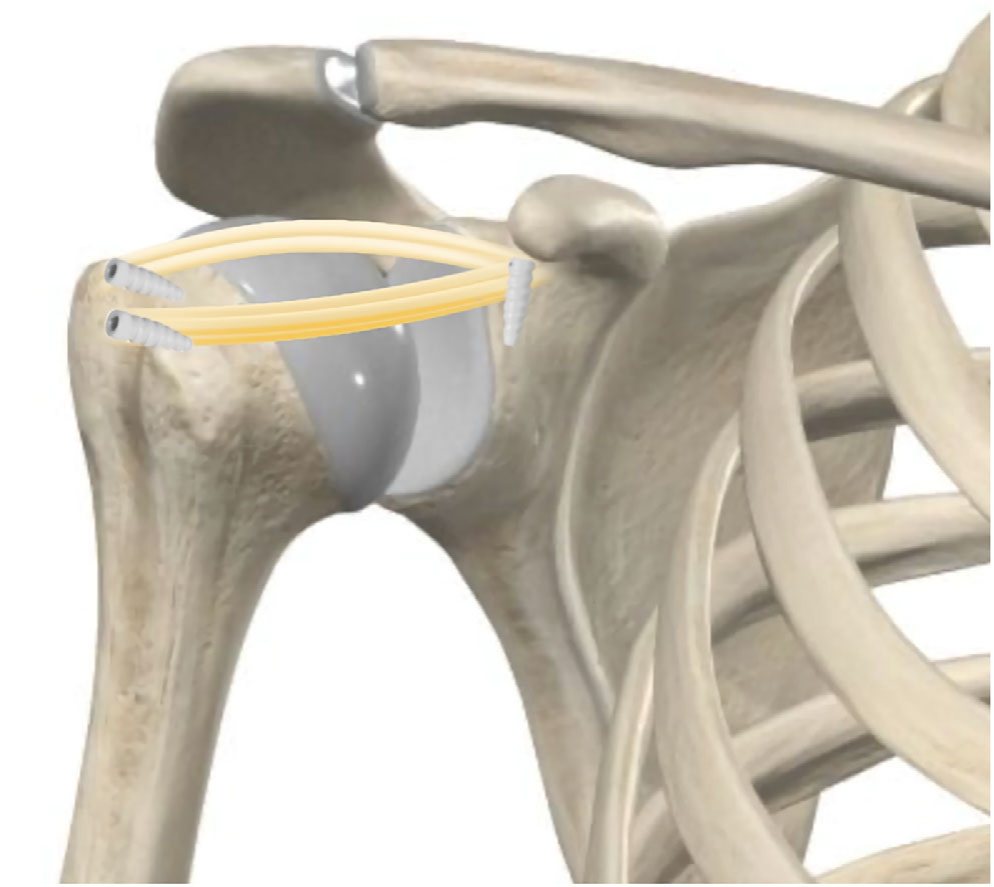
Schematic drawing demonstrating the final V‐shaped configuration of the superior capsular reconstruction using a hamstring allograft (Right side).

**FIGURE 8:**
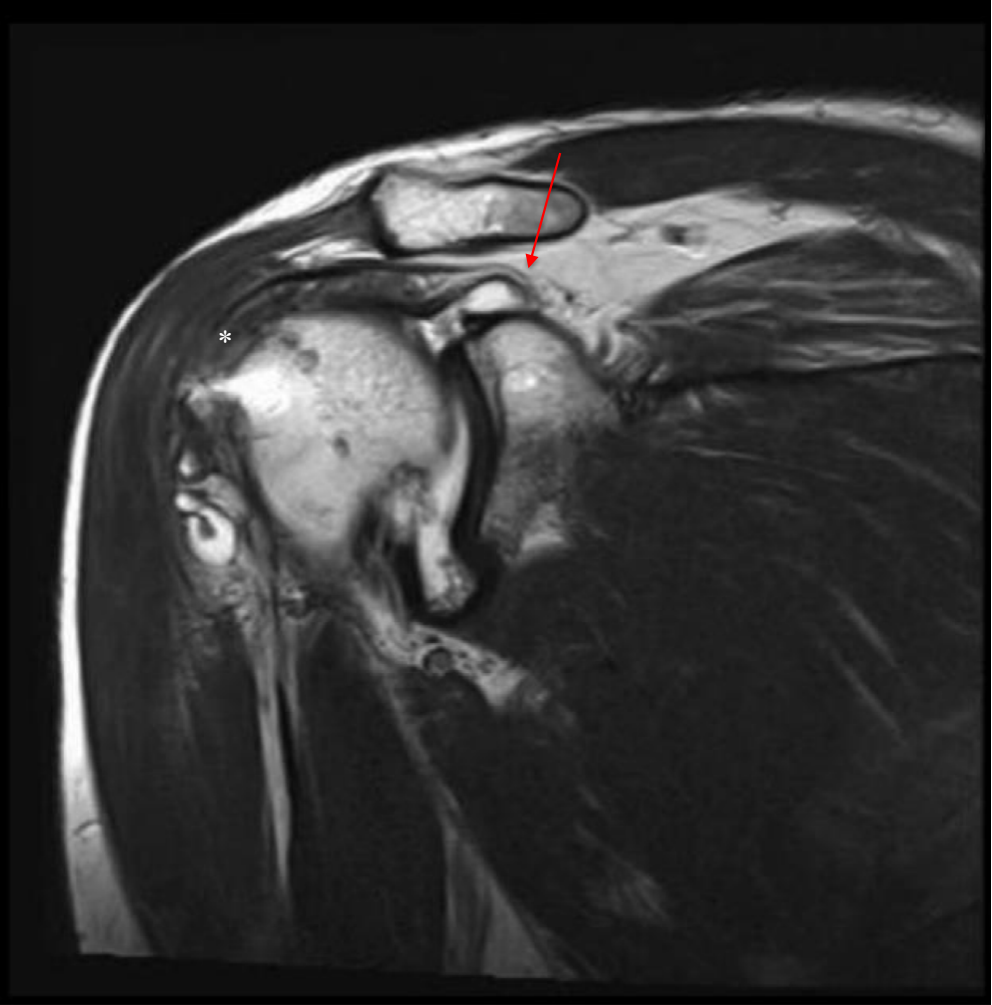
Postoperative magnetic resonance imaging at 1 year follow up shows adequate hamstring allograft integration. (arrow: glenoid border; *rotator cuff footprint).

Viewing from the posterolateral portal, insert the allograft through the lateral portal (Figure 5). Use the vertex suture for traction by retrieving it at the Neviaser portal until fully passing the tendon into the subacromial space. On the same portal, drill a pilot hole in the center of the superior border of the glenoid. Next, load the vertex suture into a 2.9 mm knotless anchor to then fix it into the pilot hole on the glenoid rim. Changing to a posterior viewing portal, retrieve the anterior strand thorough the anterolateral portal and the posterior strand through the posterolateral portal. The strands are then fixed to the anterolateral and posterolateral border of greater tuberosity using 4.5 or 5 mm knotless anchors accordingly, assuring adequate length and tension. Fixation is performed with the patient in beach‐chair position, with the operative arm maintained in longitudinal traction at 40° of forward elevation and 40° of abduction. The final construct should have a V‐shaped configuration acting as a mechanical barrier to prevent superior humeral head migration (Figures 6 and 7). Magnetic resonance imaging at 1 year follow up shows adequate allograft integration (Figure 8). Technical pearls and pitfalls are described in Table 1.

**TABLE 1:** Pearls and Pitfalls

Pearls	Pitfalls
Extensive debridement in superior glenoid rim and greater tuberosity for adequate knotless anchor placement.	Placing the pilot hole on the glenoid rim near the glenoid cartilage will result in glenoid fracture. It should be done 5 mm medial to the border.
Use smaller knotless anchors for the superior glenoid rim and thicker anchors for the greater tuberosity.	Placing the lateral fixation medial in the tuberosity may result in loosening of the allograft.
A minimum of 8 mm thickness and 10 to 12 cm graft length to prevent superior humeral head migration.	
Use of 3 high‐strength polyethylene suture (FiberWire, Arthrex, Naples, FL) for allograft preparation, 1 fir the vertex and 1 for each strand using Krackow stitch for manipulation and fixation. Prefer different color suture for better identification.	
Fixation on the greater tuberosity must be placed in the most lateral possible margin.	
Use of anterolateral and posterolateral portals for allograft fixation.	

### Rehabilitation Protocol

Initially use a shoulder abduction sling for 6 weeks. Posteriorly, passive assisted mobility should be started with a physiotherapist guidance during 4 weeks. Active mobility can be started after 10 weeks.

## DISCUSSION

Among the procedures used for treating MIRCT, we find superior capsular reconstruction, tendon transfers and partial repair. The superior capsular reconstruction, initially described by Mihata et al., using a 7.8 mm thick fascia lata autograft has shown good to excellent results at 5 years follow up, with 10% of retear rate.^5^ Thereafter, the original technique has been modified using dermal allograft, porcine dermis xenograft and, biceps tendon autograft.^6^ Even though Mihata et al. show promising results, its reproducibility is limited. Other reports such as Baek et al., describe a 62% retear rate using a 6 mm thick fascia lata autograft, without improving strength in any direction.^7^

Considering the heterogeneity in the evidence available regarding superior capsular reconstruction techniques, there are considerations to take into account. Firstly, attention must be paid to the technical difficulty of the procedure which leads to a steep learning curve in order to obtain positive results. Although multiple attempts have been made in simplify the original technique, it still represents a burden to the surgeon while perfecting its learning curve. In a cadaveric dynamic biomechanical evaluation, Berthold et al. found that using semitendinosus tendon allograft for superior capsular reconstruction in MIRCT decreases cumulative deltoid force and superior humeral head migration and increases the maximum abduction angle.^8^ These results had no significance; they represent a starting point for a more reproducible technique. Rosales‐varo et al. describes an open technique, using superior approach and hamstring graft for superior capsular reconstruction in young active patients with MIRCT reporting improvement in range of motion and an increase in acromiohumeral distance.^9^ The technique we describe for arthroscopic superior capsular reconstruction using hamstring allograft has similar biomechanical properties; it's a reproducible technique with low cost and serves as an interposition arthroplasty (Table 2). Using hamstring allograft grants the advantage of obtaining an adequate width of 8 mm. This technique aids in restoring a stable fulcrum for glenohumeral motion, which can prevent humeral head migration. The limitation to our technique is the use of frozen allograft, which can be solved by using hamstring autograft. Further studies are warranted to validate this method in larger populations at a long‐term follow up.

**TABLE 2:** Advantages and Disadvantages

Advantages	Disadvantages
Low cost and reproducible arthroscopic procedure.	Using hamstring allograft depends on its availability.
Biomechanical studies support using hamstring allograft for superior capsule reconstruction.	

## DISCLOSURES

The authors (H.C.T., V.L.J., L.A.M., R.L., G.C., R.G., F.R.) declare that they have no known competing financial interests or personal relationships that could have appeared to influence the work reported in this article.
